# Tweaking and Tweeting: Exploring Twitter for Nonmedical Use of a Psychostimulant Drug (Adderall) Among College Students

**DOI:** 10.2196/jmir.2503

**Published:** 2013-04-17

**Authors:** Carl L Hanson, Scott H Burton, Christophe Giraud-Carrier, Josh H West, Michael D Barnes, Bret Hansen

**Affiliations:** ^1^Computational Health Science Research Group, Department of Health ScienceBrigham Young UniversityProvo, UTUnited States; ^2^Computational Health Science Research Group, Department of Computer ScienceBrigham Young UniversityProvo, UTUnited States

**Keywords:** Adderall, Twitter, social media, prescription drug abuse

## Abstract

**Background:**

Adderall is the most commonly abused prescription stimulant among college students. Social media provides a real-time avenue for monitoring public health, specifically for this population.

**Objective:**

This study explores discussion of Adderall on Twitter to identify variations in volume around college exam periods, differences across sets of colleges and universities, and commonly mentioned side effects and co-ingested substances.

**Methods:**

Public-facing Twitter status messages containing the term “Adderall” were monitored from November 2011 to May 2012. Tweets were examined for mention of side effects and other commonly abused substances. Tweets from likely students containing GPS data were identified with clusters of nearby colleges and universities for regional comparison.

**Results:**

213,633 tweets from 132,099 unique user accounts mentioned “Adderall.” The number of Adderall tweets peaked during traditional college and university final exam periods. Rates of Adderall tweeters were highest among college and university clusters in the northeast and south regions of the United States. 27,473 (12.9%) mentioned an alternative motive (eg, study aid) in the same tweet. The most common substances mentioned with Adderall were alcohol (4.8%) and stimulants (4.7%), and the most common side effects were sleep deprivation (5.0%) and loss of appetite (2.6%).

**Conclusions:**

Twitter posts confirm the use of Adderall as a study aid among college students. Adderall discussions through social media such as Twitter may contribute to normative behavior regarding its abuse.

## Introduction

The mixed salt amphetamine Adderall, commonly prescribed as a treatment of Attention Deficit Hyperactivity Disorder (ADHD), is the most commonly abused prescription stimulant among college students [1]. Colleges, as well as medical and dental schools, report abuse rates of stimulant ADHD medications [2,3] ranging from a low of 8.1% to a high of 43% [4,5]. According to the National Survey on Drug Use and Health, 6.4% of college students aged 18-22 abused Adderall in the past year [6]. Given high academic expectations and competition in college settings, some students turn to prescription stimulants like Adderall as a study aid to improve concentration and increase mental alertness [7-9]. Rates of nonmedical use or abuse of ADHD drugs tend to be higher at colleges and universities where admission standards are higher [10]. A contributing factor to abuse of ADHD drugs is attention difficulties and the notion that these drugs can help with academic success [11]. DeSantis confirmed this finding and reported a higher tendency toward abuse among fraternity members during periods of high academic stress [12].  

Other studies have affirmed racial and gender discrepancies in stimulant drug abuse as well as a correlation between prescription drug abuse and other illicit drug use among college students [4,7,12,13]. Nonacademic motivations are also common and include, but are not limited to, counteracting the effects of other drugs, feeling a high, or as an appetite suppressor [7] as well as self-diagnosis of ADHD [11,14]. A contributing factor for illicit drug abuse and prescription stimulant abuse among college students is the misperception that the vast majority of their peers use drugs [15,16]. Elevated misperceptions about the prevalence of drug use among peers are attributed to the traditional media’s (eg, popular television depictions of college students using Adderall to gain academic advantages) portrayal of abuse. Misperception of reality is believed to be a leading contributor to increased levels of acceptance of abusive drug behavior, community norms for abuse, and higher levels of abuse [16]. Additional misperceptions such as the lack of danger of abusing prescription stimulants have also been found to contribute to justifications for illicit use [12].

Social media provides a relatively new and untapped resource for monitoring and understanding public health problems. As a surveillance tool, real-time data obtained through social media can be collected and analyzed quicker than traditional public health assessment tools such as questionnaires. In addition, research using social media provides an avenue for observing discussion between people in their natural interactions with one another, eliminating the Hawthorne Effect, where the presence of the researchers biases the response. Likewise, because people make statements as they occur, memory recall biases common with cross-sectional surveys or questionnaires are reduced. With the expansion of the Internet and social media, new fields of study such as infodemiology and infoveillance have emerged and represent “the science of distribution and determinants of information in an electronic medium, specifically the Internet, or in a population, with the ultimate aim to inform public health and public policy” (page 3) [17].

Studies have demonstrated the utility of online information for understanding public health problems and their determinants. Using information obtained on trends in Internet searches, researchers have predicted outbreaks of influenza [18-20], listeriosis from contaminated foods [21], and gastroenteritis and chickenpox [22]. The feasibility of using online information for epidemiological intelligence purposes has led to the creation of proprietary systems such as Google Flu Trends, which is an online search query system that has demonstrated the ability to track regional outbreaks of influenza 7-10 days in advance of conventional Centers for Disease Control and Prevention (CDC) mechanisms for reporting [23]. In addition, Healthmap represents a public system for aggregating large amounts of online information (eg, news sources) for the purpose of monitoring global disease activity [24].

Recognizing the wealth of user-generated information produced by people through their participation with social media, researchers have begun tapping or mining this information to better understand health outcomes and even health behavior. For example, Corley, Cook, Mikler, and Singh [25] mined text data in the blogosphere for “influenza” and “flu.”  Their findings revealed trends in posts about the flu that were consistent with CDC report data. Several studies mined YouTube content for information relative to immunizations [26], H1N1 influenza pandemic [27], smoking cessation [28], cardiopulmonary resuscitation [29], kidney stones [30], and prostate cancer [31]. To date, no identified study has analyzed user-generated content in social media to describe the nonmedical use of Adderall.

The purpose of this study was to leverage the power of social media (ie, Twitter) to better understand Adderall abuse as a study aid among college and university students. More specifically, the following research questions were examined: (1) When do Twitter users typically tweet about Adderall?, (2) To what extent do tweets about Adderall abuse differ among various college and university clusters in the United States?, (3) What, if any, substances do Twitter users tweet about commonly abusing in combination with Adderall?, and (4) What common side effects are mentioned?  Twitter was selected as the social media application for data collection because of its appeal with young adults including the ubiquitous research design advantages identified above. Twenty-six percent of all Internet users age 18-29 and 31% of all Internet users age 18-24 are also Twitter users [32]. Finally, using Twitter as a data source affords the ability to observe nationwide (and even international) behaviors simultaneously, as opposed to arbitrarily restricting a study to only a few regions. The use of social media data, in particular tweets, remains largely a novel concept for public health researchers. Questions surrounding the validity and utility of the data exist. Furthermore, little is known about the extent to which Twitter users might actually tweet about potentially sensitive health topics, such as Adderall abuse. Studies like the current one contribute to a type of validity testing process whereby researchers can determine the extent to which trends in Twitter content coincide with documented patterns of behavior.

## Methods

### Procedures

Twitter is a popular online social media website in which users post status updates, or “tweets,” that are limited to 140 characters. Public tweets are available and given without expectation of privacy. In addition, Twitter provides an Application Programing Interface (API), enabling programmatic consumption of the data. Specifically, the Twitter streaming API supplies tweets in real-time matching any given filter criteria. For example, using the keyword filter of “Adderall,” all tweets mentioning the substance are collected.

In addition to the content of tweets, many users also provide location indicators [33]. Specifically in Twitter, users can potentially supply exact global positioning system (GPS) coordinates (eg, from a smart phone or other GPS-enabled device) or a GPS specified place (such as a neighborhood or city). Note that users providing only state or country level GPS were not included. Furthermore, tweets were excluded if they did not originate in the United States, based on GPS location. This GPS data can be used to associate a twitter user with a nearby college/university. However, because many college campuses are within close geographic proximity, Twitter users may not necessarily have association with the campus to which they are physically nearest. Because of this proximity issue, rather than try to determine which of two nearby colleges should be used, the colleges were instead grouped into a cluster and treated as a single entity. Colleges and university with a student population of 10,000 or more were identified using the National Center for Education Statistics database. Clusters were determined using hierarchical agglomerative clustering (HAC) [34,35] with complete linkage with a cutoff distance of 150 miles. HAC produces a dendrogram of the complete sequence of nested clusterings, as follows. HAC starts by assigning each college to its own cluster. Then, the two closest clusters are merged into a single new cluster. This pairwise merging process is repeated until a single cluster containing all of the colleges is obtained. Although we have a distance defined over colleges, HAC also needs a distance over clusters. Several distance measures may be considered, the most common of which are complete linkage, which uses the maximum distance between all pairs of objects across clusters, single linkage, which takes the minimum distance, and average linkage, which computes the average of all intercluster distances. We chose complete linkage here as it tends to create more compact, clique-like clusters [36]. Given the fully nested sequence of clusterings, the choice of a specific final grouping is typically made by selecting a level at which to cut through the dendrogram, and defining the clusters as the groups of elements hanging from the subtrees whose top branches intersect with the horizontal line corresponding to the chosen level. Our cut point of 150 miles means that pairs of colleges in a cluster were no more than 150 miles apart.

The student body population of a cluster was determined by summing the populations of each included college. Twitter users are then associated with the nearest college cluster if a college in that cluster is within 100 miles of the user’s GPS location.

### Measures

Keywords related to co-ingestion with other drugs, alternative motives, and possible side effects are shown in Table 1. A case-insensitive comparison was performed to count the number of tweets containing the keywords specified. Where multiple words are given as a single term, it was considered an exact phrase.

### Data Analysis

After the tweets were obtained from the Twitter API, the data were imported to Microsoft Excel spreadsheets and then into SPSS version 20 for analysis. Frequencies, percentages, means, medians, and standard deviations were used to describe the Adderall abuse. ArcGIS 10 was used to create maps of rates for GPS Adderall tweeters.

The Institutional Review Board (IRB) at Brigham Young University approved this study.

**Table 1:** Search terms for alternative motive, co-ingestion, and side effects.

Topic	Subtopic	Search Terms
**Alternative Motive** (study aid)		test, final, finals^a^, study^a^, studi^a^, college, class, midterm, exam, homework, paper, essay, project, school, cram^a^, quiz, assignment, all-night^a^, allnight^a^
**Co-ingestion**		
	Alcohol-related	alcohol, wine, vodka, shots, patron, booz^a^, margarita, mimosa, beer, drink^a^, bud
	Stimulants	coffee, caffeine, red bull, monster, no dose, no doze, 5 hour energy, five hour energy, rockstar
	Cocaine-related	cocaine, coke, crack, rock, freebase
	Marijuana	marijuana, MJ, pot, weed, grass, reefer, Mary Jane
	Anti-anxiety	xanax, tranquilizer, valium, beanies, ativan, benzo^a^
	Meth-related	crystal, meth, methamphetamine, amphetamine
**Side Effects**		
	Sleep deprivation	tired, awake, sleep^a^, slept, insomnia, restless, asleep, trouble sleeping,
	Anxiety	anxiety, anxious, antsy, jitter^a^, shak^a^, nerv^a^, nervous, uneas^a^, worry, tense, tension, dread, restless^a^
	Teeth grinding	teeth, tooth, grind^a^, file, grat^a^, grit^a^, clench^a^, grit^a^, gnash^a^, scrap^a^
	Diarrhea	diarrhea, diarrea, diarhea, the runs, squirts
	Weakness	weak^a^, feeble, puny, scrawny
	Dizziness	dizz^a^, faint, wobbly, shaky, lightheaded, light-headed, woozy, dazed
	Headache	headache, migraine, migrain, migrane
	Sweating	sweat^a^, perspir^a^, drip^a^
	Nausea/vomiting	nausea, vomit^a^, throw up, stomach pain, stomach ache, upset stomach, puke, barf, heave
	Loss of appetite	hungry, food, eat^a^, ate, weight, appetite, meal, thin, skinny, starv^a^, slim^a^, slender
	Obsessive compulsive behavior	can't stop, clean^a^, brush^a^ teeth, wash^a^ hands, nails, nail-biting

^a^ A wildcard matching zero or more additional letters.

## Results

Using the Twitter Streaming API with the keyword filter “Adderall,” all tweets mentioning Adderall for the dates of November 29, 2011, to May 31, 2012, were collected. There were 14,282 tweets from users whose screen-names included “Adderall” or “pharm” that were removed from the sample because they were not representative of typical users, but rather those that were pushing or promoting Adderall or other pharmaceuticals. The resulting sample consisted of 213,633 tweets mentioning the term Adderall, from 132,099 unique user accounts.

The vast majority of tweets discussed Adderall use in a joking, sarcastic, or casual manner. Observed tweets included (original spelling and punctuation preserved): “I need adderall. Can't focus on studying or finishing these reviews”, “this whole no adderall for the past 3 days is really getting to me #StillDoingWork #DontKnoHowTho”, “Does anyone have adderall? #desperate”, “adderall + school = winning”, “wish i had adderall to get my room cleaned faster”, “Adderall stockpile for finals”, “We would all graduate with a 4.0 if adderall was sold over the counter”, “Running on coffee and Adderall”, “yay for adderall-induced optimism #givemeaprescription”, and “Adderall, Coffee, Red Bull. Epic focus. Or a heart attack.” Note that words beginning with “#” are hashtags, or user-defined topics that are often used in Twitter as a means of self-classification.

Table 2 lists the number of tweets matching each of the categories defined in Table 1. It should be noted that the results shown in Table 2 capture words that occur in the same tweet as the term Adderall. In this sense, they may be a conservative underestimate of actual events because it is possible that a user may tweet about Adderall but mention a side effect, motive, or another substance in another tweet. Because subtopics are not mutually exclusive, some tweets match multiple subtopics and are counted for each. Thus, the total number of *unique* tweets for a topic is not a sum of the subtopic values.

### Adderall Use by Hour, Day, and Week

**Figure 1:**
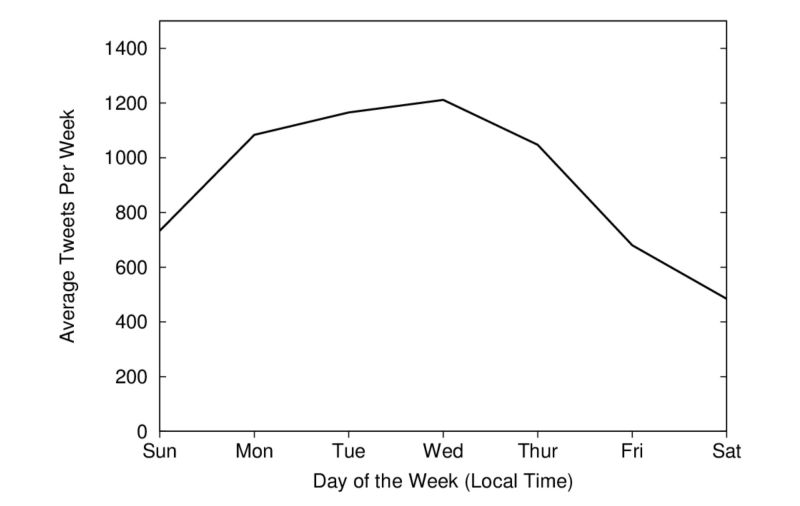
Adderall-related tweets by day of the week.

**Figure 2:**
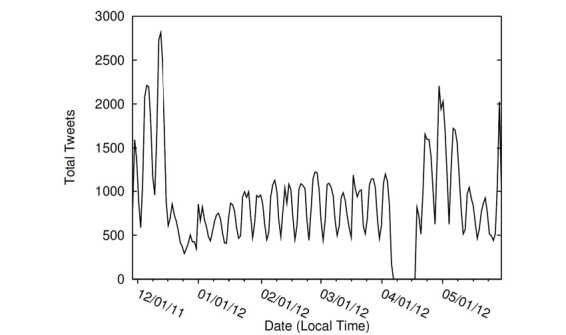
Distribution of Adderall-related tweets over 6 months.

Figure 1 illustrates the average number of Adderall-related tweets per day of the week, over the course of the study. Tweets tend to peak on Wednesday and reach a low on Saturday. As shown in Figure 2, the number of Adderall tweets per day varied significantly throughout the year, with consistently more tweets on the weekdays than the weekends. Large spikes in Twitter conversations were observed during the months of December and May—during traditional final exam times. The one-way ANOVA results indicate a significant difference between Adderall mentions between weeks (*P*<.001). Tweets regarding Adderall peaked December 13th at 2813 and April 30th at 2207 and dropped to a low of 292 on December 25th and 440 on May 27th. Over the course of 6 months while data were collected, the mean number of Adderall tweets per day was 930 with a median of 855. The large spike on May 30-31 was attributed to a US Food and Drug Administration news release warning consumers of counterfeit versions of Adderall being sold on the Internet in response to its’ being on the FDA’s drug shortage list [37]. This FDA news release was reported by news agencies, and links to the subsequent stories were tweeted by many users. The 10 days in the middle of April when no tweets were observed is the result of a failure of the investigators’ servers.

**Table 2:** Frequency distribution of Adderall tweets for search terms.

Topic	Subtopic	n	%
**Alternative Motive** (study aid)		27,473	12.9
**Co-ingestion**			
	Alcohol-related	10,229	4.8
	Stimulants	10,043	4.7
	Cocaine-related	1993	0.9
	Marijuana	1696	0.8
	Anti-anxiety	881	0.4
	Meth-related	788	0.4
	Total Unique Co-ingestion Tweets	24,167	11.3
**Side Effects**			
	Sleep Deprivation	10,687	5.0
	Anxiety	1204	0.6
	Teeth Grinding	605	0.3
	Diarrhea	11	0.01
	Weakness	140	0.07
	Dizziness	77	0.04
	Headache	223	0.1
	Sweating	381	0.2
	Nausea/vomiting	154	0.07
	Loss of Appetite	5562	2.6
	Obsessive Compulsive Behavior	1937	0.9
	Total Unique Side Effect Tweets	19,539	9.1

### College and University Clusters

Of the 213,633 tweets referencing Adderall, 27,473 (12.9%) also included reference to an alternative motive for use (eg, finals, studying, project, all-nighter), as shown in Table 2. Several of these alternative motives seem to be indicative of misuse among college-age students. To focus the analysis on college-age students, Adderall tweets were analyzed in clusters of colleges and universities that were within 150 miles of each other. A total of 586 colleges and universities in the United States were identified with a student body population of at least 10,000. Colleges and universities within 150 miles of each other resulted in a total of 87 clusters ranging in size from 1 to 48 colleges and universities in each cluster. The mean size of student-body population per cluster was 131,562, and the median was 93,281.

Of the 132,099 unique users in the sample, 3698 (2.8%) provided GPS data. In order to restrict this set of GPS-enabled users to include only those users who are likely to be students, we obtained the 3200 most recent tweets (the maximum provided by Twitter) from each user with GPS data and searched these tweets for the following student-related terms: “homework”, “teacher”, “professor”, “class”, “final”, “test”, “exam”, and “study.” Of the 3698 users with GPS information, 2335 (60.7%) included one of these student-related terms in their tweets and are referred to as GPS Adderall Tweeters.

**Figure 3:**
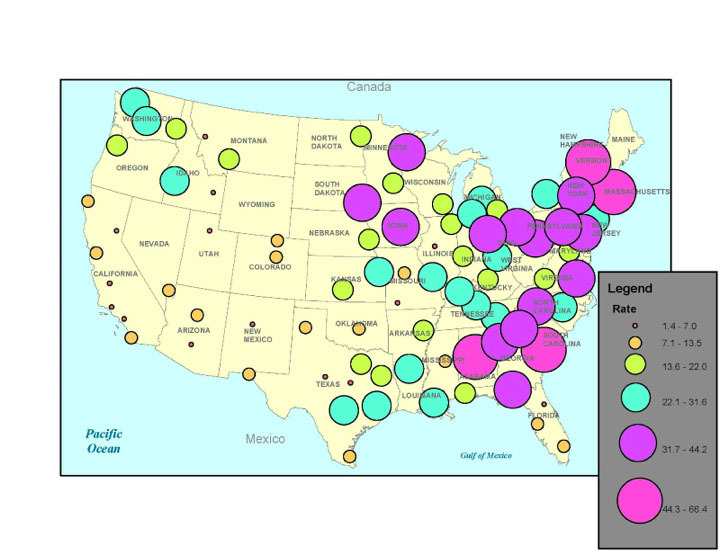
Rates of Adderall tweets by 150 mile college clusters in the United States (rate per 100,000 students).

Figure 3 illustrates the 150-mile college clusters in the contiguous 48 states of the United States according to the rate of GPS Adderall Tweeters per 100,000 students, where the center of the circle is the average of the locations of the colleges in the cluster, and the size of the circle corresponds to the rate. Table 3 lists the ten clusters with the highest rates, and Table 4 lists the ten clusters with the lowest rates. Cluster identifications (ID) represent the state(s) to which the majority of colleges and universities in the cluster belong. As shown in these tables, the amount of GPS Adderall Tweeters per 100,000 students ranges from a high of 66.4 in the Vermont cluster and 54.6 in the Massachusetts cluster to a low of 1.4 in the South-Eastern Texas cluster and 2.1 in the Central Illinois cluster. Rates reveal a greater rate of GPS Adderall Tweeter in the northeast and south regions of the United States.

**Table 3:** Top 10 rates of Adderall tweets for 150-mile college and university clusters in the United States.

Rank	ID^a^	Rate	GPS Adderall Tweeters	Total Cluster Population	Number in the Cluster
1	Vermont	66.4	9	13,554	1
2	Massachusetts	54.6	162	296,704	16
3	Alabama	52.2	38	72,748	3
4	South Carolina, Southern Georgia	48.8	57	116,891	6
5	Central Georgia	44.2	52	117,765	6
6	North Georgia, Southern South Carolina	44.0	36	81,773	4
7	Northern Florida	44.0	18	40,921	3
8	Southern Pennsylvania, Northern West Virginia	42.9	43	100,336	5
9	Ohio	37.3	54	144,659	7
10	Western North Carolina, Eastern Tennessee	37.0	41	110,718	6

^a^ ID represents the state(s) to which the majority of colleges and universities in the cluster belong.

**Table 4:** Bottom 10 rates of Adderall tweets for 150-mile college and university clusters in the United States.

Rank	ID^a^	Rate	GPS Adderall Tweeters	Total Cluster Population	Number in the Cluster
77	Central Texas	5.8	3	52,076	3
78	Alaska	5.5	1	18,154	1
79	Southern California	5.5	55	1,008,210	48
80	Puerto Rico	5.0	3	60,579	4
81	Northern Nevada	4.5	3	66,242	4
82	New Mexico	3.9	3	77,236	3
83	Northern Utah, Southern Idaho	3.6	1	27,476	1
84	Northern California	3.5	4	115,026	7
85	Central Illinois	2.1	1	46,797	3
86	South-Eastern Texas	1.4	1	69,949	3

^a^ ID represents the state(s) to which the majority of colleges and universities in the cluster belong.

### Co-ingestion and Side Effects

A total of 19,125 (8.9%) tweets also mentioned another substance along with Adderall in their tweet (see Table 2). Analysis revealed that the most common substance terms were alcohol-related (4.8%, n=10,229) and stimulants, such as coffee or Red Bull (4.7%, n=10,043). Other substances were cocaine-related (0.9%, n=1993), marijuana (0.8%, n=1696), methamphetamine-related (0.4%, n=788), and depressants, such as Xanax and painkillers (0.3%, n=728).

Sleep deprivation (5.0%, n=10,687) and loss of appetite (2.6%, n=5562) were the most common side effects associated with Adderall tweets (see Table 2). Diarrhea (0.01%) was the least common side effect mentioned followed by weakness (0.01%, n=140) and nausea/vomiting (0.07%, n=154).

## Discussion

This study demonstrated the use of Twitter posts (ie, tweets) as a way to examine Adderall abuse among a sample of college students in the United States. More specifically, the study sought to determine: (1) When do Twitter users typically tweet about Adderall?, (2) To what extent do tweets about Adderall abuse differ among various college clusters in the United States?, (3) What, if any, substances do Twitter users tweet about commonly abusing in combination with Adderall?, and (4) What common side effects are mentioned?

Findings indicate that Twitter posts regarding Adderall vary across day of the week and week of the month. Consistent with traditional college final exams schedules, tweets regarding Adderall peaked during December and May. Similarly, tweets regarding Adderall peaked during the middle of the academic week and declined to fewer mentions over the weekend. These findings are consistent with previous research that has suggested that college students who abuse prescription ADHD stimulants do so primarily during times of high academic stress [12]. In addition, preexisting attention difficulties have been shown to be a predictor of nonmedical use of prescription ADHD medication in order for college students to experience greater academic success [11].

Grouping colleges within 150-mile clusters ultimately provided a mechanism for comparing geographic regions within the United States. Analysis of these college clusters revealed a concentration of GPS Adderall tweeters along the northeastern portion of the United States and in some of the southern states. The rates of GPS Adderall twitters per 100,000 students in the east and south clearly indicated greater Twitter conversations related to the use and abuse of Adderall. These findings are consistent with previous studies that examined the nonmedical use of prescription stimulants. McCabe [4] observed geographical patterns of nonmedical use of prescription stimulants with higher rates of use among college students in the north-eastern region of the country. Additionally, these findings are consistent with the Monitoring the Future study where higher rates of nonmedical methylphenidate use were found among college-age young adults in the northeastern region of the United States [38]. Other studies at select colleges in the east have shown high rates of nonmedical use of prescription stimulants [8,9].

Additional research is needed to better understand the reasons for geographical variations in use. One possible explanation includes the fact that the U.S. fraternity/sorority system has deep historical roots at northeastern colleges and universities, and prevalence of nonmedical use of prescription stimulants is higher among fraternity/sorority members [4]. Future research might explore the link between nonmedical use of prescription stimulants and the geographical distribution of colleges and universities and their admission standards, student/family income, as well as the distribution of prescription drug monitoring program in the United States. Research has associated nonmedical use of prescription stimulants with competitive admission standards [4] and students coming from families with higher incomes [39].

Geographical findings can provide practitioners with evidence necessary for prioritizing intervention resources for targeting priority populations. This study has demonstrated how grouping can occur; however, and more importantly, it provides a social media solution for segmenting a broader population into more meaningful and manageable groups for intervention purposes. Colleges can be clustered in numerous different ways as needed and defined by researchers.

Because social media is, by its very nature, a *social* endeavor, the users’ postings can have a great impact on the social norms of others. This is particularly relevant in the context of drug abuse, where drug abuse behavior can be represented. Social norms theory suggests that individual behavior (eg, drug use) is influenced by individual perceptions of what is perceived as “normal” or “typical.” This theory is rooted in Social Cognitive Theory [40] as well as the Theory of Planned Behavior [41]. In this light, the data that 8.9% of Adderall tweets mention another substance in the same tweet is significant because it may influence others to think that co-ingestion is normal and not dangerous. This is particularly troubling because it is through poly drug use or co-ingestion that morbidity and mortality risk increases. Poly drug use occurs among college Adderall abusers and combining Adderall with other stimulants like cocaine increases risk of heart attack and stroke [6]. Also in this regard, even tweets that are sarcastic, joking, or simply restating song lyrics, are relevant in their misrepresentations because of their impact on social norms.

Nearly 1 in 10 tweets included in this sample referenced a side effect of Adderall use/abuse. Effects relative to sleep deprivation and loss of appetite were discussed the most. Whereas more tweeters discussed an alternative motive for use (ie, study aid), individual tweeter perception of the benefits of Adderall use (eg, study aid) may outweigh the costs of use (eg, side effect such as irritability). Future research might further explore individual perceptions of Adderall side effects among college students to gain a better understanding of why some college students abuse, while others do not.

### Limitations

These findings should be interpreted based on the following limitations. First, not every Adderall tweet is related to actual use. For example, we observed song lyrics that impact these counts, such as the two often quoted lines “College hoes love alcohol and popping adderall” and “I’ve been up for 3 days… adderall and redbull.” In our sample, there were 4275 tweets that have the words “college hoes love” and 894 that have the words “been up for three/3 days”. These numbers likely inflate the number of matches for “college”, “alcohol”, and “redbull” above the number of people tweeting about actually using these substances. However, as discussed, even sarcastic mentions, or the quotation of song lyrics, are pertinent because of the impact they may have on social norms. Second, our study did not consider misspellings of the word “Adderall” or other ADHD medications, such as Ritalin. While our sample would have been increased by these inclusions, it is not likely that their absence resulted in any particular sampling bias. Third, our analysis focused exclusively on public tweets. It is unclear, and indeed difficult to assess, what the impact of other tweets (eg, direct messages) may have on our results. Fourth, our analysis focused only on colleges and universities with a student population of 10,000 or more. No attempt was made to designate whether the colleges and universities in this sample were on a quarter or semester system. Finally, the keyword approach to identifying college students may have included other students (eg, high school) or others that simply mentioned academic-related terms. While these additional users could inflate our overall values, we have no reason to believe they would be substantially biased toward different areas of the nation.

### Conclusions

The twitter-based surveillance methodology in this study produced similar findings to traditional survey designs. In response to the noted research questions, Twitter posts regarding Adderall vary across day of the week and week of the month among users. Consistent with college traditional final exams schedules, tweets regarding Adderall peaked during December and May. Similarly, tweets regarding Adderall peaked during the middle of the academic week and declined to fewer mentions over the weekend, which suggests that college students who abuse prescription ADHD stimulants do so primarily during times of high academic stress.

Additionally, tweets about Adderall abuse differ among various college clusters in the United States. Using 150-mile college clusters, regional comparisons identified a concentration of GPS Adderall tweeters along the northeastern portion of the United States and in some of the southern states, and thus indicate greater Twitter conversations related to the use and abuse of Adderall. Further, co-ingestion of other substances, notably alcohol, stimulants (such as coffee or Red Bull), cocaine-related, marijuana, methamphetamine-related, and depressants, (such as Xanax and painkillers) are the substances most commonly mentioned with Adderall. Such poly drug use or co-ingestion is known to increase morbidity and mortality risk. Finally, the most common side effects associated with Adderall tweets include sleep deprivation and loss of appetite. Thus, Adderall abuse is associated with college or university life. Given the risks and trends for Adderall acceptance among college-age students, there is a need to renew interest and priorities to influence college campus norms, promote the safe and legal use of these substances, and promote stronger student wellbeing and study habits to better manage the academic demands and pressures that are typical on college campuses in the United States.

## Abbreviations

API: Application Programming Interface
CDC: Centers for Disease Control and Prevention
GPS: Global Positioning System
HAC: Hierarchical Agglomerative Clustering
ID: Identification
IRB: Institutional Review Board
